# Monthly Summary

**Published:** 1869-08

**Authors:** 


### MONTHLY SUMMARY.

*Excision of Inferior Dental Nerve for Neuralgia.*—Wm. J. C. æt. 22. This case is one of a very interesting character. The patient has had a violent form of facial neuralgia for three years. He has suffered great agony from the excruciating pain. Every attempt at talking, mastication, or deglutition, is attended with an aggravation of the symptoms. His life has become burdensome. His lips and cheeks are in constant spasmodic action. The expression of the countenance is denotive of pain. The angle of the mouth is constantly drawn over to the right side. The pain is more particularly severe along the right side of the upper and lower lips. Both the upper and lower teeth were extracted, without even temporary benefit. The pain is most severe when he eats. It very often wakes him at night. It is worse when the weather is wet. Every medical measure has been tried in vain.

As the probability is that the pain is confined chiefly to the branches of the inferior dental nerve, it is proposed to make an incision along the course of that nerve, curvilinear in shape, carrying it nearly as far as the symphysis of the chin, and to perforate the bone with a trephine, making thus three, four, or five openings, and then to take out this nerve in its entire length. Prof. Gross, has performed this operation in a number of cases, and in all of them with very decided relief. Inasmuch as all the ordinary remedies against neuralgia have been tried in this case without any avail at all, the probability is that the immediate cause of the difficulty is pressure exerted upon the nerve in the long canal through which it passes. The nerve itself is probably not diseased, but simply encroached upon in the manner indicated. In performing the operation, the inferior maxillary artery will, of course, be divided. This will be ligated, and the flaps dissected up to expose the surface of the bone. Then, by means of the trephine, the external plate of the jaw-bone will be divided, and thus admission obtained to the seat of the disease.

The patient was placed under the influence of chloroform, and the operation performed in the manner detailed. The wound was dressed with the twisted suture.

Nov. 7th. The patient has had no neuralgic pain since the operation. There is a slight erysipelatous blush in the neck, which was painted with dilute tincture of iodine. The extirpated nerve has been microscopically examined, and no evidence of disease detected in its structure. It is therefore probable that the stream of nerve fluid was interrupted by some pressure upon the nerve, giving rise to the neuralgia. *Surgical Clinic of Prof. Gross.—Med. & Surg. Reporter.*

---

*Treatment of Diarrhœa.*—Dr. Wm. Mason Turner, of Philadelphia, reports, in the *Medical and Surgical Reporter*, that in the beginning of the diarrhœa season, the past summer, he used successfully, calomel and opium in decided doses, and in nine hours after the commencement of this, seidlitz powder, rochelle salt or castor oil; and after two good stools showing the action of the mercury, he gradually checked the bowels with vegetable astringents, mustard or spice plasters to the abdomen, and ice in small quantities. Later, fancying he saw deleterious effects from the calomel (his patients seemed suddenly to grow weak under its use) he immediately discontinued it, and without using the *mercurial*, or the *saline and anodyne* treatment, resorted *at once* to the vegetable astringents. He gave immediately, from three to four drachms of a mixture of equal parts of tr. opii, camphor, tr.camph., tr. zingib., tr. krameriæ and tr. lav. com., and in "an hour after, gave every two hours, half tea spoonful doses of above for six or eight times." By this method, he says, he treated some very bad cases, and with the most satisfactory results. "The bowels invariable are promptly checked; one day after the diarrhœa has ceased, open the bowels with some ol. olivæ, or the castor oil mixture. This practice may not be orthodox, but the stools are healthy and remain so; and orthodox or not, it is successful."

---

*Consumption of Horse-Meat in Paris and Berlin.*—The sale of horse-meat has not taken so well in Paris as might have been expected from the first success of the undertaking. We are informed by the official accounts, which have just been published, that during the last twelve months the number of horses slain in Paris amounts to 2400. Out of this quantity five per cent. have been employed in making sausages, etc., whilst forty per cent. have been sold to the small *restaurants*, and ten per cent. to the poorer classes. It may thus be seen that the quantity of horse-meat knowingly consumed as such in Paris is very small. Indeed, even the poorest people in that city manifest a strong aversion to horseflesh.

The number of horses slain in Berlin during the same period of time amounts to 4044, thus forming almost double the number slaughtered in Paris. But it may be well to add that the Berlin dyers are now making an extensive use of horse-blood.—*London Lancet.*

---

*To Prevent Death by Chloroform.*—Experiments on inferior animals show that they may be restored from apparent death from chloroform by the continuous galvanic current, the negative pole being put in the mouth and the positive pole in the rectum. In some cases the animal was left for two minutes in a state of apparent death and then restored.—*Med. Record.*

*Salivary Calculus from Wharton's Duct.* By John L. Firestone, M. D.—A farmer, æt. 50, called on me with a tumor in the floor of the mouth, under the right side of the tongue, which has been noticed a "good while," as it interfered somewhat with mastication, though not with deglutition.

On examination a hard tumor was felt in the place designated, apparently about the size of a hulled walnut, and on the floor of the mouth Wharton's duct was seen unusually open. A probe was easily introduced, and immediately below the surface struck a hard, rough calculus. Introducing a narrow-bladed bistoury and opened the sac parallel with the tongue, and with the scoop end of a director, I removed a calculus 14 lines long; 8 broad and 6 deep, and 1½ drachms in weight. It was yellowish-white; oval, with flattened sides; and was composed of "phosphate and carbonate of lime, held together by animal matter."

This salivary calculus is probably of unusual size. Rokitansky says, referring to these formations, that they vary in size "from a millet-seed or a pea, to even that of a hazel-nut."—*Detroit Review of Med.*

---

*Coffee as a Deodorizer.*—Coffee is spoken of in high terms by foreign journals as a deodorizer for the neutralizing of foul odors that emanate from organic bodies in a state of decay, as it can be used to advantage where other disinfecting agents would be inadmissable. In cases where rats die in the spaces between the floors of dwellings, the intolerable odor arising therefrom can be effectually removed by placing a pound or two of fresh burnt and ground coffee between the floors. For the purification of a sick room it is incomparably superior to burning rags, as it has a beneficial chemical action on the atmosphere of the room, and gives, besides, an agreeable perfume.

---

*Iodine and Carbonic Acid for Local Application.*—As a substitute for this, Dr. Alex. Boggs reccommends in the *Lancet* the following: "Tincture of iodine, one drachm and a half; glycerine, two ounces; solution of chlorinated lime, six ounces. Half an ounce of this solution to six or eight ounces of water is used in all cases to which the tincture of iodine or chlorinated lime is applicable. The solution is perfectly colorless, and may be used with advantage as an injection in diseases with fetid discharges." *Am. Eclectic Med. Review.*

